# Risk Assessment of *Anopheles philippinensis* and *Anopheles nivipes* (Diptera: Culicidae) Invading China under Climate Change

**DOI:** 10.3390/biology10100998

**Published:** 2021-10-03

**Authors:** Chao Li, Yuan Gao, Nan Chang, Delong Ma, Ruobing Zhou, Zhe Zhao, Jun Wang, Qinfeng Zhang, Qiyong Liu

**Affiliations:** 1 School of Public Health, Shandong First Medical University, Jinan 250000, China; lichaoicdc@163.com (C.L.); madelong97@163.com (D.M.); 2 State Key Laboratory of Infectious Disease Prevention and Control, Collaborative Innovation Center for Diagnosis and Treatment of Infectious Diseases, National Institute for Communicable Disease Control and Prevention, Chinese Center for Disease Control and Prevention (China CDC), Beijing 102206, China; gaoyuancdc@126.com (Y.G.); changnan@njmu.edu.cn (N.C.); zrb9610@126.com (R.Z.); zhezhao@mail.sdu.edu.cn (Z.Z.); wangjun@icdc.cn (J.W.); 3 School of Public Health, Nanjing Medical University, Nanjing 210000, China; 4 Shandong University Climate Change and Health Center, School of Public Health, Shandong University, Jinan 250012, China

**Keywords:** *Anopheles*, climate change, potential suitable areas, risk assessment

## Abstract

**Simple Summary:**

Climate change has a significant impact on the quantity and distribution of vectors and may thus threaten the health of the population. We used the maximum entropy model to predict the near-current distribution of potentially suitable areas for *Anopheles philippinensis* and *Anopheles nivipes* in the world, as well as the distribution of potentially suitable areas in China under future climate scenarios. We also constructed a vector risk assessment system to assess the possibility of the two mosquito species invading China. Among the meteorological factors, the precipitation in September makes the greatest contribution to the distribution of areas suitable for the two mosquito species, which have a moderate risk of invading China. The relevant management departments should formulate scientific prevention and control measures for the two mosquitoes according to meteorological factors and the risk level of invasion.

**Abstract:**

Background: *Anopheles philippinensis* and *Anopheles nivipes* are morphologically similar and are considered to be effective vectors of malaria transmission in northeastern India. Environmental factors such as temperature and rainfall have a significant impact on the temporal and spatial distribution of disease vectors driven by future climate change. Methods: In this study, we used the maximum entropy model to predict the potential global distribution of the two mosquito species in the near future and the trend of future distribution in China. Based on the contribution rate of environmental factors, we analyzed the main environmental factors affecting the distribution of the two mosquito species. We also constructed a disease vector risk assessment index system to calculate the comprehensive risk value of the invasive species. Results: Precipitation has a significant effect on the distribution of potentially suitable areas for *Anopheles philippinensis* and *Anopheles nivipes*. The two mosquito species may spread in the suitable areas of China in the future. The results of the risk assessment index system showed that the two mosquito species belong to the moderate invasion risk level for China. Conclusions: China should improve the mosquito vector monitoring system, formulate scientific prevention and control strategies and strictly prevent foreign imports.

## 1. Introduction

Global climate change has a profound effect on natural ecosystems [1]. According to the fifth assessment report of the United Nations Intergovernmental Panel on Climate Change (IPCC AR5), global land and ocean temperatures rose by 0.89 °C (0.69~1.08 °C) from 1901 to 2012. Climate change will alter the suitable range of species [2], which is directly related to biological invasion, and may expand the negative impact of alien species in many areas [3].

Malaria is a vector-borne disease caused by *Plasmodium* spp. and is endemic in 102 countries and regions, which are mainly located in Africa, Southeast Asia, and Central and South America. The pathogens (etiological agents, *Plasmodium* parasites) are transmitted through the bite of *Anopheles* mosquitoes [4,5]. According to the World Malaria Report 2020, there are nine malaria-endemic countries in the Southeast Asian region, accounting for about 3% of the global morbidity burden of malaria, of which India accounts for 88%. In India, in addition to the three main malaria vectors, *Anopheles baimaii* (formerly *An. dirus* species D), *An. minimus* s.l. and *An. fluviatilis* s.l., there aresome other mosquito vectors, such as *Anopheles philippinensis* and *Anopheles nivipes*, which are considered to be potential vectors of malaria parasites in this area [6,7,8].

Both *An. philippinensis* and *An. nivipes* belong to the subgenus *Cellia*, *Anopheles annularis* group [9]. They are very similar in morphology, and it is difficult to distinguish them as adults. Therefore, researchers usually refer to them as the *Anopheles philippinensis-nivipes* complex [10]. Molecular-based research has clarified the potential importance of the *Anopheles philippinensis-nivipes* complex in the transmission of malaria, which has aroused the WHO’s attention to the spread of *Plasmodium falciparum* and/or *Plasmodium vivax* in the states of northeastern India [10,11,12,13]. Northeast India borders China. China has a vast territory, a complex geographic environment, and increasingly frequent trade exchanges, which greatly increases the spread risk of *An. philippinensis* and *An. nivipes*. Once they invade China without any precautionary measures in place, the risk of malaria transmission willincrease. Therefore, it is necessary to assess the invasion risk of *An. philippinensis* and *An. nivipes* to China.

The maximum entropy model (MaxEnt v3.4.1 (Robert Schapire’s Home Page; http://rob.schapire.net/; accessed on 29 March 2021.)) is a species distribution prediction model based on the maximum entropy theory and has shown a strong predictive ability among many species distribution prediction models [14], especially when the species distribution point data are insufficient; it is often better than other analogous prediction models [15,16,17,18,19,20]. We imported into the model the current distribution point data of the species and the environmental variable data required for prediction, and simulated the possible distribution of the target species by calculations [21]. In this study, the MaxEnt model was used to predict the areas potentially suitable for *An. philippinensis* and *An. nivipes* under current and future climatic conditions. Then we constructed a vector risk assessment index system, and evaluated the invasion risk of *An. philippinensis* and *An. nivipes* into China from three aspects, including invasion risk, colonization and spread risk as well as damage effect. The results could provide a basis for the relevant departments to develop effective surveillance measures and reasonable prevention and control policies.

## 2. Materials and Methods

### 2.1. Data Collection and Preprocessing

#### 2.1.1. The Selection of Occurrence Points

**Figure 1:**
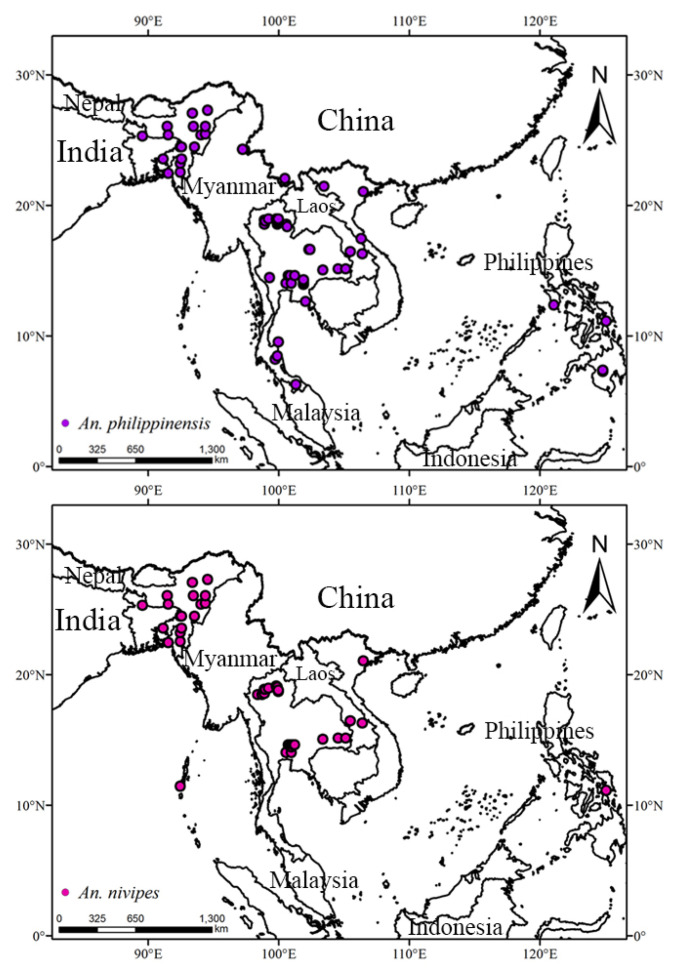
Global distribution of *Anopheles philippinensis* and *Anopheles nivipes*.

The occurrence points of *An. philippinensis* and *An. nivipes* were acquired mainly from the Global Biodiversity Information Facility(GBIF; GBIF.org (5 March 2021) GBIF Occurrence Download https://doi.org/10.15468/dl.mhtg6g and GBIF.org (7 May 2021) GBIF Occurrence Download https://doi.org/10.15468/dl.xmzmd9) database and related research literature of CNKI, Web of Science, PubMed, MEDLINE, Embase, and other databases. We obtained 80 and 53 valid records of *An. philippinensis* and *An. nivipes*, respectively. By valid records, we mean that we established strict inclusion criteria for the selection of distribution sites of the two mosquito species. First of all, when we downloaded the distribution points from the GBIF database, we deleted the duplicate distribution points and the distribution points without accurate information (such as the exact location, the missing person who uploaded the data, andserious location deviation). Secondly, when collecting the literature from databases with the key words [*Anopheles philippinensis* or *Anopheles nivipes*] AND [Climate change or Global warming or Climate scenarios] AND [Monitor or Distribution or ENMs or SDMs], we selected the literatures with exact geographical location (as accurate as possible below the county) and specific mosquito species and population density information in the monitoring data of the two mosquito species. Then, we used Google Maps to represent the collected distribution points in latitude and longitude (Tables S3 and S5). In addition, ArcGIS was used to display all the collected data on the world map, deleting points that deviated significantly from the actual points (such as in a certain sea area). We also used the ENMTOOLs to keep only one distribution point in the same grid based on the 5 km × 5 km meteorological data grid and deleted the redundant distribution points to avoid overfitting of the results [22]. We used the above steps to obtain valid records. Finally, we organized the collected results into CSV files (Tables S4 and S6) of species name, longitude and latitude, which were used to import the MaxEnt model. After screening and processing, 59 distribution sites of *An. philippinensis* and 42 distribution sites of *An. nivipes* were preserved (Figure 1).

#### 2.1.2. Environmental Variables

Environmental variables were collected from Worldclim (http://www.worldclim.org/; accessed on 1 June 2021) with a spatial resolution of 5 arc-minutes, including bioclimatic variables (bio1-bio19), monthly maximum temperature (tmax1–tmax12), monthly minimum temperature (tmin1–tmin12), monthly precipitation (prec1–prec12) and elevation (ele). We imported environmental data under the near-current climate scenario (1970–2000) and future climate scenarios (2021–2040, 2041–2060, 2061–2080, 2081–2100) into the model, which included four climate scenarios (ssp126, ssp245, ssp370 and ssp585) of the BCC-CSM2-MR global climate model, which represents different social sharing economic paths of future climate change. The downloaded data were transformed into ASC format which could be recognized by MaxEnt software through ArcGIS (version10.6; Esri, Redlands, CA, USA).

To avoid the overfitting of environmental variables, the Pearson correlation analysis was used to analyze the correlation between the environmental variables using R (version4.0.3; https://www.r-project.org/; accessed on 1 June 2021) software. Variables with the absolute value of correlation coefficient more than 0.8 were regarded as highly correlated, and variables with the absolute value of correlation coefficient less than 0.8 were retained. According to the results of the first run of the MaxEnt model, variables with the highest contribution rate were included in the model, and variables with less than 1% contribution rate were deleted. Finally, seven variables were selected as predictors of *An. philippinensis*, and four variables were selected as predictors of *An. nivipes* (Table 1).

#### 2.1.3. Map Data

We obtained a digital map of China (scale: 1:4,000,000) from National Geomatics Center of China (http://www.ngcc.cn/ngcc/; accessed on 30 May 2021) and downloaded the distribution map (scale: 1:10,000,000) of the world from Natural Earth (https://www.naturalearthdata.com/downloads/; accessed on 30 May 2021).

### 2.2. Methods

#### 2.2.1. Prediction of Areas Suitable for *Anopheles philippinensis* and *Anopheles nivipes*

MaxEnt was used to predict the areas suitable for *An. philippinensis* and *An. nivipes* under near-current and future climate scenarios. We used ArcMap software, DIVA-GIS software v7.5.0 (https://www.diva-gis.org/; accessed on 4 March 2021), and R software to calculate the regularization multiplier (RM). The packages, including ENMeval, dismo, dotCall64, fields, grid, knitr, maps, maptools, raster, rgeos, sp, spam, and spTh in R software were used to optimize the model parameters [23]. The Akaike information criterion(AIC) value in the ENMeval package was used to measure the goodness of the statistical model fit, and a lower AIC value was preferred. According to the test, the best parameter combination for *An. philippinensis* was Q (quadratic), H (hinge) and P (product) with the RM value of 4. Similarly, the best parameter combination for *An. nivipes* was L (linear), Q (quadratic) and H (hinge) with the RM value of 2.5. The distribution points and environmental variables were imported into the model with 25% of the distribution points as the test set. The model was run with the selected optimal parameters. The prediction results were all output in ASC grid data format with values ranging from 0 to 1. The predicted grid value was converted into the species-suitable area level by using the ArcMap reclassification module. This study used the lowest presence threshold (LPT) to define the suitable distribution area and unsuitable distribution area [24]. The contents of the potential distribution areas were divided into four categories including non-suitable areas (0–LPT), poorly suitable areas (LPT–0.4), moderately suitable areas (0.4–0.6), and highly suitable areas (0.6–1.0).

#### 2.2.2. Risk Assessment of *Anopheles philippinensis* and *Anopheles nivipes* Invading China

This risk assessment system comprehensively analyzes the possibility of invasion and colonization of invasive species, and their impact on the environment and society, by referring to the main pest risk assessment index system [25]. The system integrated the source and consequence factors that could promote the formation of alien species invasion risk in the whole process of invasion with a starting point of whether the vector could invade mainland China (Table S1). The risk assessment index system is divided into four levels. The first level is the target level, which is expressed by the comprehensive risk index (R). It describes the final result of the risk assessment of the invasion of vectors into China. The second level is the project level. According to the general process of vector invasion, namely “invasion risk, colonization and diffusion risk, and damage effect”, all risk factors affecting vector invasion are divided into three categories, including introduction risk (P), colonization and diffusion risk (E) and damage risk (I). The third level is the factor level, which determines the risk factors of each project level. The fourth level is the index level, which is the specific index to describe each evaluation factor. To facilitate a unified calculation, according to the principle of fuzzy mathematics, referring to cases of biological invasion risk assessment [25,26], the specific value of the index that can be quantified was set, and the index that cannot be quantified was evaluated and scored by experts according to their own experience. The R value is determined by the risk of *P*, *E* and *I*. The logical relationship between them conforms to the principle of multiplication. The calculation formula can be expressed as Equation (1).

(1) $$R=\sqrt[{3}]{P\times E\times I}$$

The calculated comprehensive risk value should be between 0 and 1. The contents of comprehensive risk value were divided into five categories including very low (0–0.2), low (0.2–0.4), moderate (0.4–0.6), high (0.6–0.8), and very high (0.8–1.0). According to the algorithm principle, the calculation formula of *P*, *E* and *I* is expressed as Equations (2)–(4)

(2) $$P=\sqrt[{t}]{\prod {Ρ}_{i}}\;(i=3),$$

(3) $$E=\sqrt[{t}]{\prod E_{i}}\;(i=7)$$

(4) $$I=\max\left(I_{i}\right),$$

The input pathway (*P*_1_) and the evaluation indexes *P*_11,_ *P*_12_ and *P*_13_ conform to the multiplication principle. Therefore, the calculation formula is given as Equation (5).

(5) $$P_{1}=\sqrt[{3}]{P_{11}\times P_{12}\times P_{13}},$$

$E_{2,},E_{6}$ and $E_{7}$ are in accordance with the additive relationship with the corresponding indicators, and Equation (6) is the calculation formula is Equation (6) ($\omega_{Ai}$ is the weight value of the corresponding index, *n* is the corresponding index level, and *A* = 2, 6, 7). The weight coefficient $\omega_{Ai}$ is determined by the analytic hierarchy process (AHP).

(6) $$E_{A}=\sum_{i=1}^{n}\omega_{Ai}E_{Ai}/\sum \omega_{Ai},$$

## 3. Results

### 3.1. Prediction of Suitable Areas for Anopheles philippinensis and Anopheles nivipes

#### 3.1.1. Suitable Areas under Near-Current Climate Scenarios

**Figure 2:**
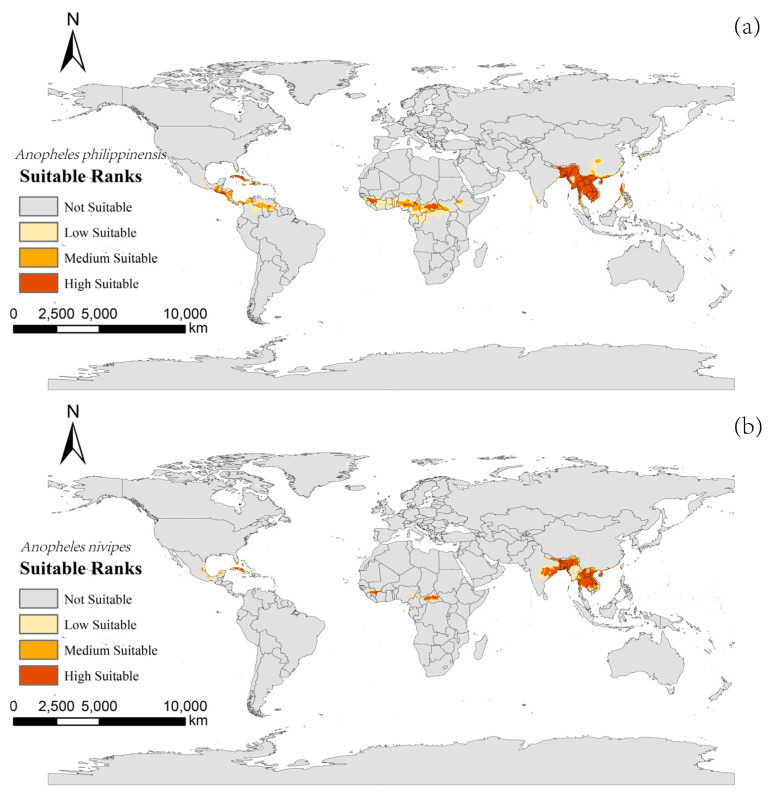
Potentially suitable areas for *Anopheles philippinensis* (**a**) and *Anopheles nivipes* (**b**) at the current time using the MaxEnt model.

The potentially suitable areas for *An. philippinensis* under near-current climate scenarios were mainly located in Asia (including Nepal, Bhutan, Bangladesh, Myanmar, Vietnam, Laos, Thailand, Cambodia, Philippines and the southern border of China), Africa (including Guinea, Sierra Leone, Nigeria, Cameroon and the Central African Republic), North America (including Nicaragua, El Salvador, Honduras and Cuba), and South America (Venezuela). The areas suitable for *An. nivipes* were mainly located in Asia (including Nepal, eastern India, Bangladesh, Vietnam, Laos, Thailand, Cambodia, Philippines, and the southern border of China), Africa (including Guinea and Central African Republic), and North America (Cuba and western Mexico; Figure 2).

#### 3.1.2. Suitable Areas in China under Future Climate Scenarios

**Figure 3:**
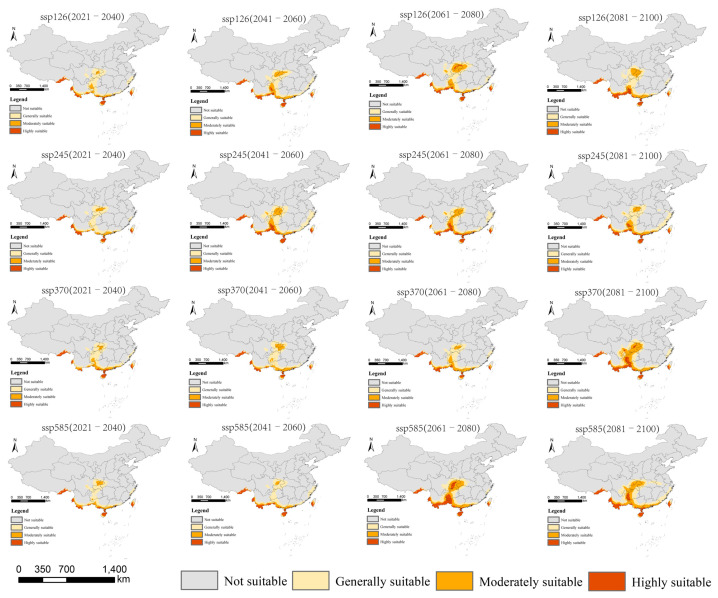
Potentially suitable areas for *Anopheles philippinensis* at the future climate scenarios using the MaxEnt model.

**Figure 4:**
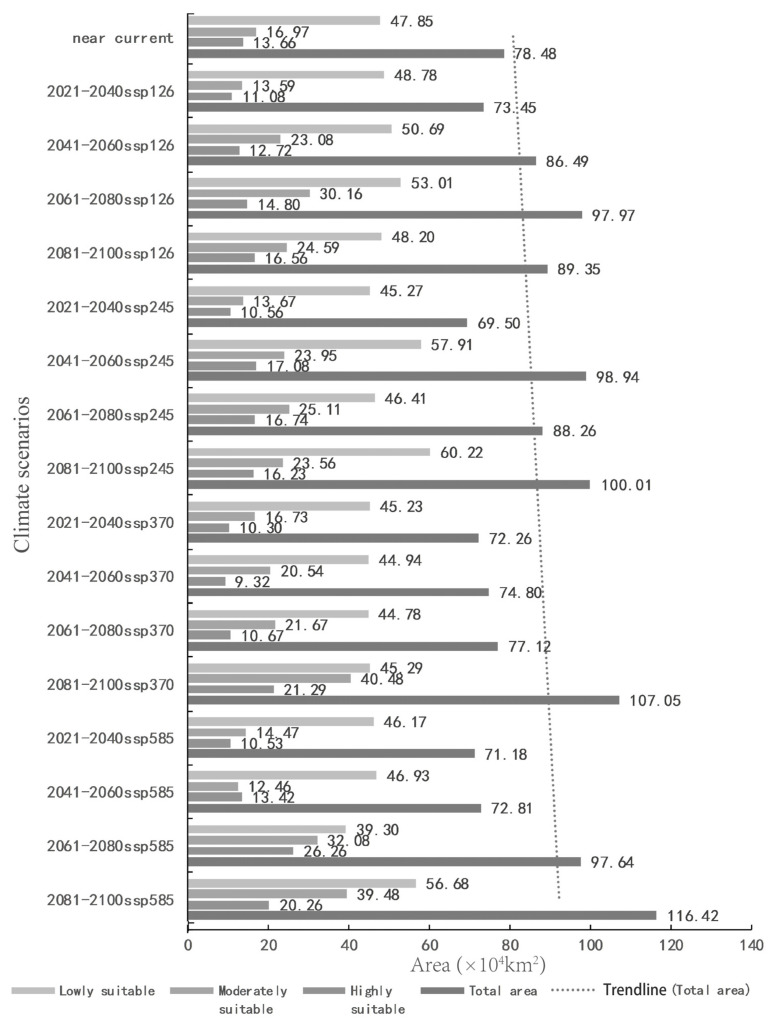
Change trend of potentially suitable areas for *Anopheles philippinensis* in China under near-current and future climate conditions.

**Figure 5:**
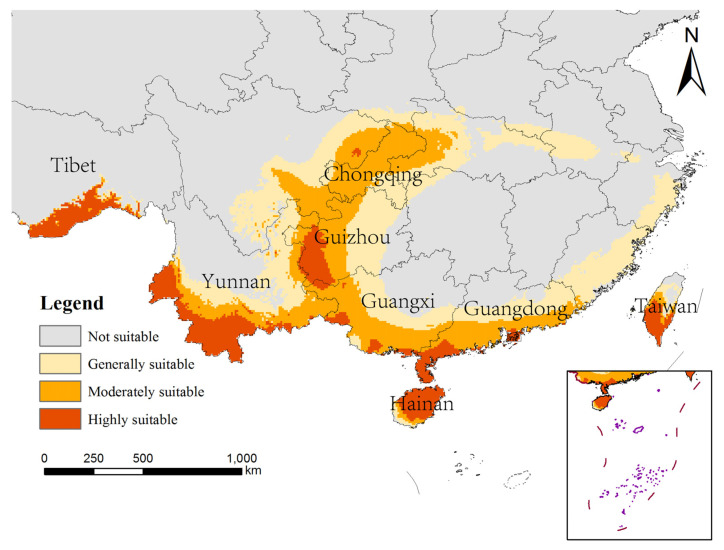
Distribution of potentially suitable areas for *Anopheles philippinensis* in China under SSP585 climate scenarios in 2081–2100s.

According to the prediction results for different periods and climate scenarios in the future, we extracted the suitable areas for *An. philippinensis* and *An. nivipes* in China. The potentially suitable areas for *An. philippinensis* under future climate scenarios in China are mainly distributed along the southwest border of Tibet and in southern Yunnan, southern Guangxi, southern Guangdong, Hainan, Chongqing, Guizhou and central and southern Taiwan. Moreover the area potentially suitable for *An. philippinensis* under various climate scenarios in the future shows an overall increasing trend (Figure 3 and Figure 4), especially under the SSP585 climate scenario from 2081 to 2100, *An. philippinensis* had the most suitable area in China (Figure 5).

**Figure 6:**
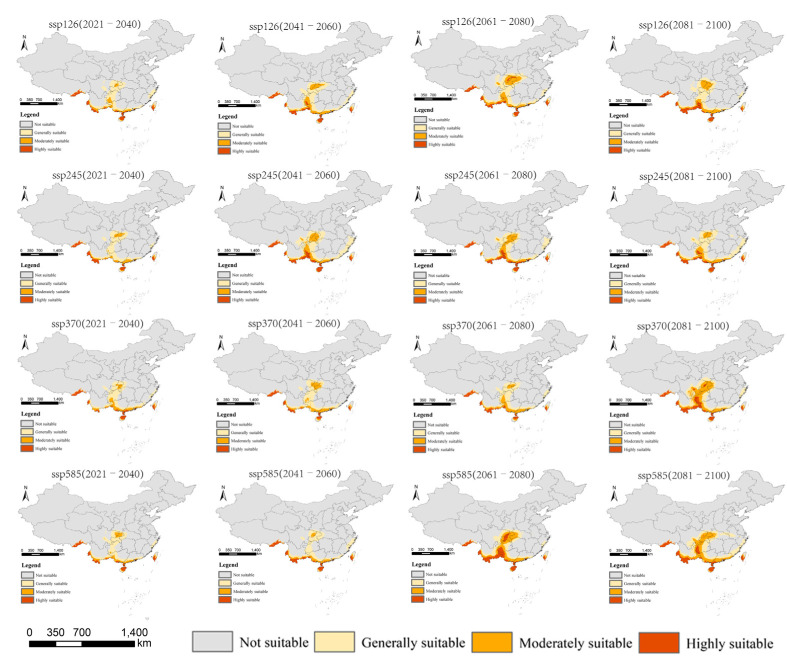
Potentially suitable areas for *Anopheles nivipes* in future climate scenarios using the MaxEnt model.

**Figure 7:**
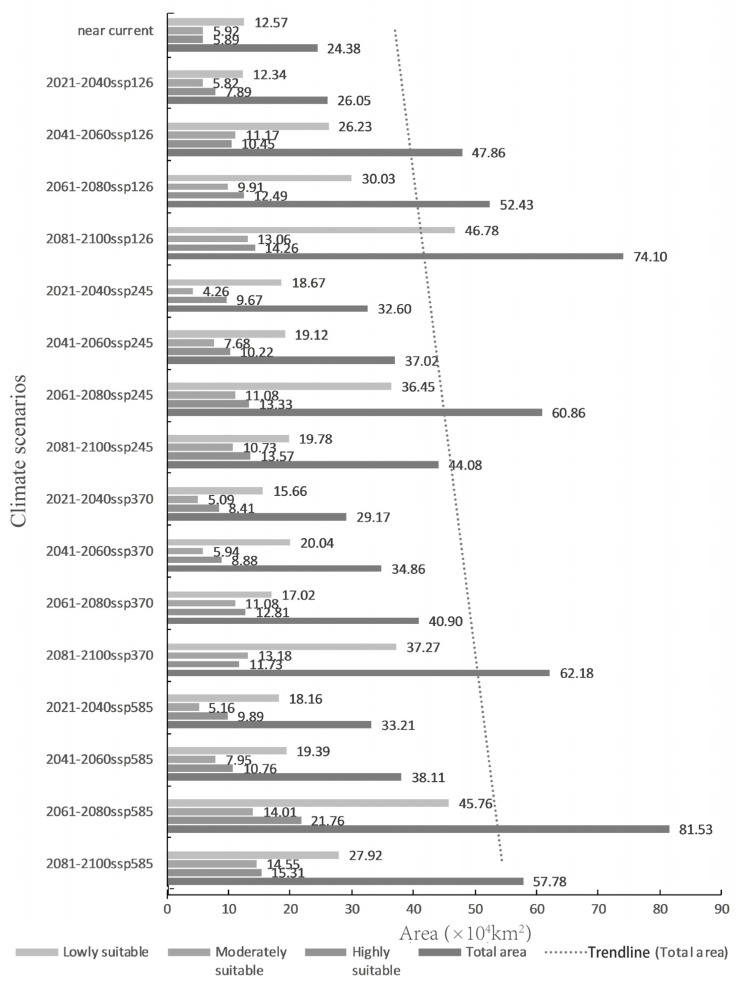
The changing trend of potentially suitable areas for *Anopheles nivipes* in China under near-current and future climate conditions.

**Figure 8:**
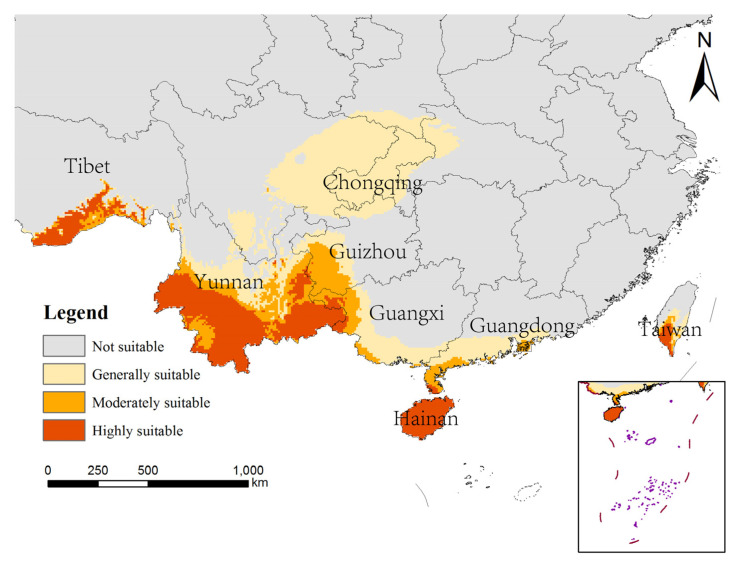
Distribution of potentially suitable areas for *Anopheles nivipes* in China under SSP585 climate scenarios in 2061–2080s.

The potentially suitable areas for *An. nivipes* under future climate scenarios in China are mainly distributed along the southwest border of Tibet and in southwestern and southeastern Yunnan, southern Guangxi and Guangdong, Hainan and the south-central part of Taiwan. The area of potentially suitable for *An. nivipes* under various climate scenarios in the future shows an overall increasing trend (Figure 6 and Figure 7), especially under the SSP585 climate scenario from 2061 to 2080; the area suitable for *An. nivipes* has the largest range in China (Figure 8).

### 3.2. Risk Assessment Results

By consulting experts on biological invasion and consulting the literature, we obtained the risk assessment index level scores of *An. philippinensis* and *An. nivipes* (Table S2). The comprehensive risk value of *Anopheles philippinensis* and *Anopheles nivipes* was 0.49 and 0.44, respectively, which belong to the moderate risk invasive species (Table 2).

### 3.3. The Predictive Accuracy of the Maximum Entropy Model

**Figure 9:**
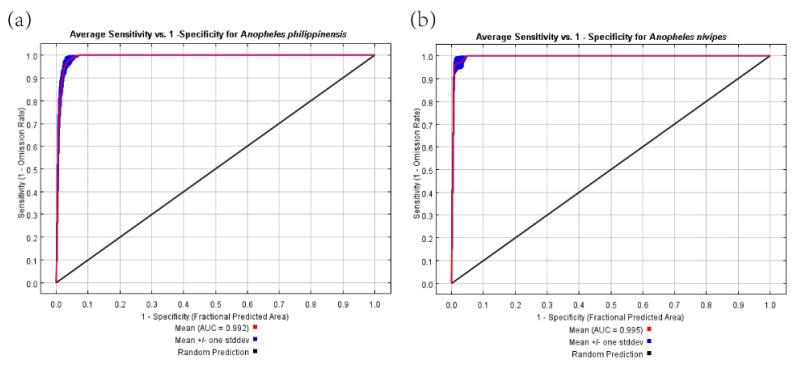
The AUC values predicted by the maximum entropy model for *Anopheles philippinensis* (**a**) and *Anopheles nivipes* (**b**).

Based on the data of occurrence points of species and environmental variables, we used the maximum entropy model to predict the worldwide potential distribution area of *An. philippinensis* and *An. nivipes*. The accuracy of the predicted results was estimated using the AUC value. The AUC value of *An. philippinensis* and *An. nivipes* was 0.992 and 0.995, respectively (Figure 9), which showed that the accuracy of the model is very high.

## 4. Discussion

In this study, we found that areas highly suitable for *An. philippinensis* and *An. nivipes* are along the southern border of China. Under the climate scenario of the 21st century, the highly suitable areas may spread to former unsuitable areas and moderately suitable areas. As vectors threatening human health, *An. philippinensis* and *An. nivipes* have been monitored in Yunnan and Hainan, China in recent years. Combined with the analysis of geographical location, climate conditions, and the southwest monsoon of the Indian Ocean and the Himalayas, northeast India has become one of the regions with the highest rainfall in the world, which further proved the rationality of prec9 as the environmental factor with the highest contribution rate to the distribution of *An. philippinensis* and *An. nivipes*. Considering future climate change and the suitability of temperature and rainfall for the survival of mosquitoes, the risk of mosquito spread and breeding would increase along the southern border of China, which would further endanger the health of people.

### 4.1. Relationship between Environmental Variables and Potential Spread of Anopheles philippinensis and Anopheles nivipes

We found that the most important environmental variable affecting the potential distribution of *An. philippinensis* and *An. nivipes* is precipitation in September (prec9), which contributes most to the survival suitability of these two species. Then, the order in which environmental factors influence the distribution of the suitable area of *An. philippinensis* according to contribution rate is as follows: precipitation in May (prec5), precipitation seasonality (bio15), precipitation of the coldest quarter (bio19), precipitation in December (prec12), precipitation in March (prec3), and temperature seasonality (bio4), respectively. By contrast, the order in which environmental factors influence the distribution of *An. nivipes* is as follows: precipitation of the coldest quarter (bio19), precipitation in March (prec3), temperature seasonality (bio4) and altitude (ele). By observing the distribution records of *An. philippinensis* and *An. nivipes*, we found that the longitude and latitude of the distribution points of the two species were roughly the same, and they were located in the tropical monsoon climate area with abundant rainfall, which was consistent with our findings that precipitation has a great influence on the survival of *An. philippinensis* and *An. nivipes*. 

### 4.2. Invasion Risk and Control Suggestions of Anopheles philippinensis and Anopheles nivipes under Future Climate Conditions

Comparing the range of potentially suitable areas for the two mosquito species in China under future climate conditions with that under near-current climate conditions, the total suitable area for the two mosquito species showed an increasing trend. The total suitable area for *An. philippinensis* increased the most under the SSP585 climate scenario for the period 2081–2100, and the predicted total suitable area increased from 78.48 × 10^4^ km^2^ to 116.42 × 10^4^ km^2^. Under SSP585 climate scenario for the period of 2061–2080, the total suitable area for *An. nivipes* increased the most, and the predicted total suitable area increased from 24.38 × 10^4^ km^2^ to 81.53 × 10^4^ km^2^. 

Referring to the rainfall data of China in recent years, we found that the eastern part of the Sichuan Basin in the upper reaches of the Yangtze River has abundant rainfall in September every year. The prediction of the maximum entropy model showed that this region is moderately and highly suitable for *An. philippinensis* and *An. nivipes* under some future climate scenarios. Mosquito monitoring data in recent years have shown that *An. philippinensis* and *An. nivipes* are found in the Lancang River Basin and the Hainan Island in China. Moreover, we creatively constructed the risk assessment index system of vector biology and the reference standard of index assignment to comprehensively evaluate the invasion risk of the two mosquito species. The above research results show that *An. philippinensis* and *An. nivipes* are moderately invasive risk species for China. That is to say, the tow species have high potential invasion harmfulness in China, so it is necessary to strengthen monitoring measures to prevent further spread.

Affected by the future global climate change, *An. philippinensis* and *An. nivipes* are likely to spread to inland China, so we need to be vigilant against import. Although local malaria cases in China have been reported for three consecutive years, the situation of cases imported from abroad continues to pose a risk. We suggest that for the prevention and control of the spread of malaria vector mosquitoes, we take the following measures: (1) control the breeding of mosquitoes and remove their larvae and eggs from riverbeds and ponds around September, because Prec9 has made an important contribution to the distribution of mosquitoes; (2) improve the construction of mosquito-monitoring and early-warning platforms and grasp the dynamics of the mosquito population. Especially in areas that are moderately or highly suitable for the two mosquitoes, mosquito surveillance and investigation should be strengthened; (3) further assess the risk of malaria and malaria vectors with a multi-model system because the impact of climate change on malaria is very important, ; (4) actively deal with imported cases to prevent malaria from spreading again.

### 4.3. Advantages and Limitations

The advantage was that the simulation accuracy of the maximum entropy model is greater than 0.9, and we also used the ENMeval datapacket to optimize the RMvalue. Generally, AUC values greater than 0.9 indicated that the model has high accuracy (Figure 9). On the other hand, we applied the updated Worldclim database, which contains the latest version of current and future representative environmental data. The prediction results have a certain reference significance for the use of different versions of climatic data. 

To increase the reliability of the distribution points used in the study, we not only deleted the duplicate distribution points and the distribution points with serious lack of information, but also further screened the distribution points. First, we checked the specific source of the sample geographic coordinates obtained in the GBIF database. If the coordinate point information came from the molecular database, it was verified by the molecule. Second, the coordinate points from the literature in some GBIF databases provide specific references, so we download the corresponding references to check whether the samples were verified by molecules. Similarly, when collecting species distribution points in the process of literature retrieval, we also reviewed the relevant literature in detail and excluded the literature that did not identify species accurately [27]. Finally, we gave priority to selecting the distribution sites of the two mosquito species with molecular identification results, and excluded the literature that failed to accurately identify the two mosquito species and the distribution sites with unknown species information.

However, our study has some limitations . Firstly, the climate data used in our study were from 1970 to 2000 to simulate t near-current climate conditions. There is still a 20-year gap from the current timetable, which means that there are still limitations in predicting potential areas for the species because researchers do not have access to the latest climate database. Secondly, wrong species identification information affects the accuracy of the prediction results of the model. Considering the morphological similarity of the two mosquito species, it is very difficult to accurately identify the species in the field. Although specific information about mosquito species is given in some references, the researchers in that literature may not have made an accurate molecular identification of the species, thereby resulting in misrecognition. All this these will affect the reliability of the geographical distribution points of the two mosquito species used for model training. Thirdly, we used only the maximum entropy model to predict the potentially suitable habitat for the two species; other species distribution models, such as GARP and CLIMEX, would be considered in our next step to provide more evidence.

This study constructed a vector biological risk assessment index system based on the clear vector *Anopheles* mosquitoes. The highly suitable area and comprehensive risk value of invasive species delineated by the maximum entropy model and the risk assessment index system can be used as a reference for risk assessment of other species. Through the comprehensive analysis of the invasion risk, colonization and diffusion risk and damage effect of the species, this risk assessment index system involves not only the biological characteristics of the species, but also ecological adaptability, human controllability, social and public health hazards and so on. Although many related indicators are involved, they are still not enough to represent the true evaluation results of species. In future research, further optimization needs to be performed in the selection of indicators.

## 5. Conclusions

In this study, we used the maximum entropy model to predict the distribution of potentially suitable areas for *An. philippinensis* and *An. nivipes* under the near-current and future climate scenarios. The area potentially suitable for *An. philippinensis* and *An. nivipes* under various climate scenarios in the future shows an overall increasing trend. The results showed that the two species may spread in suitable areas of China in the future and that rainfall would have a great influence on the suitable areas. Combined with the vector risk assessment index system, we found that the two species are moderately invasive due to many indicators, such as characteristics of the species and the impact of future climate change. China needs to formulate targeted early-risk management measures to achieve early detection and early management so as to prevent the spread of the two mosquito species.

## Figures and Tables

**Table 1:** Percentage contributions and permutation importance of the bioclimatic variables included in the Maxent models for *Anopheles philippinensis* and *Anopheles nivipes*. Variables with contribution less than 1 (indicated by ×) were removed because of high cross-correlations.

Symbol	Bioclimatic Variables	*Anopheles philippinensis*		*Anopheles nivipes*	
		Contribution (%)	Permutation Importance	Contribution (%)	Permutation Importance
Prec9	Precipitation inSeptember	46.7	6	71	43.5
Prec5	Precipitation in May	35.1	64.8	×	×
Bio15	Precipitation Seasonality	7.5	0.1	×	×
Bio19	Precipitation of Coldest Quarter	5.9	2.6	17.2	14.6
Prec12	Precipitation in December	2.2	0.4	×	×
Prec3	Precipitation in March	1.6	2	6.9	6.9
Bio4	Temperature Seasonality	1	24.2	4.3	32.9

**Table 2:** Project level and comprehensive risk value of risk assessment indicators of *An. philippinensis* and *An. nivipes*.

Species	Project Level			Comprehensive Risk Value	Invasion Risk Level
	Introduction Risk (P)	Colonization and Diffusion Risk (E)	Damage Effect (I)		
*An. philippinensis*	0.47	0.47	0.50	0.49	Moderate
*An. nivipes*	0.42	0.41	0.51	0.44	Moderate

## Data Availability

Bioclimatic data used in this study can be downloaded at https://www.worldclim.org/; accessed on 1 June 2021. Occurrence records used in this study can be downloaded at https://www.gbif.org; accessed on 7 May 2021.

## Supplementary Materials

The following supplementary materials are available online at https://www.mdpi.com/2079-7737/10/10/998/s1. Table S1: Risk assessment index system of vector biology and the reference standard of index assignment; Table S2: Risk assessment results of *An. philippinensis* and *An. nivipes*; Table S3: Distribution points of *An. philippinensis* for model operation; Table S4: Information on the distribution points of *An. philippinensis* collected through a literature search; Table S5: Distribution points of *An. nivipes* for model operation; Table S6: Information on the distribution points of *An. nivipes* collected through a literature search.
