# Supplementary material for: Risk Assessment of Anopheles philippinensis and Anopheles nivipes (Diptera: Culicidae) Invading China under Climate Change
Source: Biology (Basel). 2021 Oct 3;10(10):998. doi: 10.3390/biology10100998 (PMC8533129; doi:10.3390/biology10100998)
Supplement: Supplementary file 1 [file biology-10-00998-s001.zip › Table S1.pdf]

Table S1 Risk assessment index system of vector biology and reference standard of index assignment.

Project level	Factor level	Index level	Indicator assignment reference standard				
			0	0<x≤0.25	0.25<x≤0.5	0.5<x≤0.75	0.75<x≤1.00
Introduction risk (P)	Introduction possibility (P_1)	Percentage of existing distribution countries in the world. (P_{11})	/	0<Proportion≤25%	25%<Proportion≤50%	50%<Proportion≤75 %	Proportion>75%
		Introduction frequency of vector (P_{12})	Times per year = 0	Times per year<4	4≤Times per year<12	12≤Times per year<24	24≤Times per year<48
		Invasion probability with natural vector or disperse (P_{13})	Natural dispersal cannot occur	/	/	/	Natural dispersal occur
	Survival risk(P_2)	Survival rate during transportation process P_{21})	The survival rate = 0	0<The survival rate≤10%	10%<The survival ratee≤20%	20%<The survival rate≤50%	50%<The survival rate≤100%
	Barrier function(P_3)	Quarantine efficiency P_{31})	Very high	High	Moderate	Low	No
Colonization and diffusion risk(E)	Distribution of potential suitable areas(E_1)	Proportion of potential suitable areas for vector organisms in invading countriesE_{11})	/	0<Proportion≤25%	25%<Proportion≤50%	50%<Proportion≤75 %	Proportion>75%
	Reproduction ability (E_2)	Reproduction generation (E_{21})	Cannot be passed on from generation to	One generation per year	Two generation per year	Three or four generation per year	More than four generation per year
		Reproduction mode (E_{22})	Insect cannot reproduce with parthenogenesis	/	/	/	Insect can reproduce with parthenogenesis
		Maximum fecundity of one femaleE_{23})	/	0<Reproduction quantity≤50	50<Reproduction quantity≤100	100<Reproduction quantity≤200	Reproduction quantity>200
	Climatic conditions (E_3)	Suitability of abiotic factors e.g. temperature, light, precipitations etc(E_{31})	Not suitable	Slightly suitable	Moderately suitable	High suitable	Very high suitable
	Substrate conditions (E_4)	Survival suitability in varied food and hostE_{41})	0	0<Suitability degree≤10%	10%<Suitability degree≤50%	50%<Suitability degree≤80%	80%<Suitability degree≤100%
	Tolerance to stresses (E_5)	Tolerance to adverse conditions (E_{51})	None	Weak	Moderate	High	Very high
	Dispersal ability (E_6)	Ability of active flight and passive disperse with natural vectors (E_{61})	None	Weak	Moderate	High	Very high
		Frequency of propagule dispersed with human activities e.g. transportation, tourism etc E_{62})	None	Low	Moderate	High	Very high
	Control effect (E_7)	Control effects of biotic factors (E_{71})	Very effective	Effective	Moderate	Low	Very low
		Monitoring difficulty E_{72})	Very effective	Easy	Moderate	Difficulty	Very difficulty
		Eradication and control difficulty E_{73})	Very easy	Easy	Moderate	Difficulty	Very difficulty
Damage effect (I)	Social and public health risk(I_1)	Impacts on landscape aesthetic values (I_{11})	Very low	Low	Moderate	High	Very high
		Impacts on politics, culture and religions etc I_{12})	Very low	Low	Moderate	High	Very high
		Impacts on human health, wild and domestic animals etc (I_{13})	Very low	Low	Moderate	High	Very high
		Impacts on resident life and sequence work (I_{14})	Very low	Low	Moderate	High	Very high
	Ecological risk (I_2)	Impacts on soil, atmosphere, surface and ground water, microclimate I_{21})	Very low	Low	Moderate	High	Very high
		Impacts on interspecific interaction of native species and biodiversity (I_{22})	Very low	Low	Moderate	High	Very high
		Ability to act as vectors carrying with other pathogens (I_{23})	Very low	Low	Moderate	High	Very high
	Economic risk (I_3)	Economic loss to the treatment of crowd diseases (I_{31})	Very low	Low	Moderate	High	Very high
		Economic losses to directly related industries I_{32})	Very low	Low	Moderate	High	Very high
		Economic losses to other indirect aspects(I_{33})	Very low	Low	Moderate	High	Very high
