# Supplementary material for: Risk Assessment of Anopheles philippinensis and Anopheles nivipes (Diptera: Culicidae) Invading China under Climate Change
Source: Biology (Basel). 2021 Oct 3;10(10):998. doi: 10.3390/biology10100998 (PMC8533129; doi:10.3390/biology10100998)
Supplement: Supplementary file 1 [file biology-10-00998-s001.zip › Table S2.pdf]

Table S2. The two mosquito species risk assessment index level scores

	An. philippinensis					*An. nivip*		
Index leve	Expert evaluation score				Average score	Expert evaluation scor		
P_{11}	0.1	0.07	0.2	0.6	0.2425	0.06	0.05	0.2
P_{12}	0.8	0.2	1	1	0.75	0.8	0.1	1
P_{13}	1	1	0.8	0.2	0.75	1	1	0.8
P_{21}	0.75	0.7	0.6	0.2	0.5625	0.7	0.5	0.5
P_{31}	0.7	0.5	0.2	0	0.35	0.6	0.6	0.2
E_{11}	0.15	0.15	0.2	0.3	0.2	0.1	0.1	0.2
E_{21}	1	0.3	0.2	0.8	0.575	1	0.2	0.2
E_{22}	0.22	1	0.2	0	0.355	0.21	0.2	0.2
E_{23}	1	1	1	0.2	0.8	1	1	1
E_{31}	0.23	0.3	0.8	0.3	0.4075	0.2	0.25	0.8
E_{41}	0.75	0.3	0.7	0.3	0.5125	0.7	0.25	0.7
E_{51}	0.25	0.65	0.6	0.3	0.45	0.22	0.35	0.5
E_{61}	1	0.65	0.7	0.2	0.6375	1	0.35	0.6
E_{62}	0.7	0.1	0.4	0.1	0.325	0.68	0.1	0.4
E_{71}	0.2	0.25	0.2	1	0.4125	0.18	0.35	0.2
E_{72}	0.75	0.25	0.7	0.2	0.475	0.75	0.25	0.7
E_{73}	1	0.65	0.7	0.6	0.7375	1	0.7	0.7
I_{11}	0.5	0.5	0.7	0.2	0.475	0.4	0.5	0.7
I_{12}	0.45	0.5	0.2	0.1	0.3125	0.4	0.5	0.2
I_{13}	0.68	0.7	0.5	0.1	0.495	0.66	0.5	0.5
I_{14}	0.26	0.7	0.2	0.1	0.315	0.25	0.5	0.2
I_{21}	0.1	0.75	0	0.2	0.2625	0.1	0.75	0
I_{22}	0.2	0.75	0.3	0.2	0.3625	0.15	0.75	0.3
I_{23}	0.1	0.5	0.4	0.1	0.275	0.1	0.2	0.4
I_{31}	0.15	0.75	0.7	0.4	0.5	0.15	0.75	0.75
I_{32}	0.1	0.2	0.5	0.2	0.25	0.08	0.2	0.5
I_{33}	0.25	0.2	0.4	0.2	0.2625	0.23	0.2	0.4

es

e	Average score
0.5	0.2025
1	0.725
0.2	0.75
0.1	0.45
0	0.35
0.2	0.15
0.8	0.55
0	0.1525
0.2	0.8
0.4	0.4125
0.3	0.4875
0.3	0.3425
0.2	0.5375
0.1	0.32
1	0.4325
0.2	0.475
0.6	0.75
0.2	0.45
0.1	0.3
0.1	0.44
0.1	0.2625
0.2	0.2625
0.2	0.35
0.1	0.2
0.4	0.5125
0.2	0.245
0.2	0.2575
