# Supplementary material for: Risk Assessment of Anopheles philippinensis and Anopheles nivipes (Diptera: Culicidae) Invading China under Climate Change
Source: Biology (Basel). 2021 Oct 3;10(10):998. doi: 10.3390/biology10100998 (PMC8533129; doi:10.3390/biology10100998)
Supplement: Supplementary file 1 [file biology-10-00998-s001.zip › Table S3.pdf]

Species	Longitude	Latitude	Location	Country	Reference
Anopheles	92.4431	22.5333	Mizoram	India	Sarma NP, Prakash A, Bhattacharyya DR, Kali
Anopheles	93.3635	27.0512	Arunacha	India	Sarma NP, Prakash A, Bhattacharyya DR, Kali
Anopheles	94.3531	26.0709	Assam	India	Bhattacharyya DR, Prakash A, Sarma NP, Mol
Anopheles	94.4047	25.4318	Arunachal	India	Bhattacharyya DR, Prakash A, Sarma NP, Mol
Anopheles	93.3635	27.0512	Meghalaya	India	Bhattacharyya DR, Prakash A, Sarma NP, Mol
Anopheles	92.4304	24.4344	Mizoram	India	Bhattacharyya DR, Prakash A, Sarma NP, Mol
Anopheles	92.5109	23.5246	Nagaland	India	Bhattacharyya DR, Prakash A, Sarma NP, Mol
Anopheles	91.1645	23.5009	Tripura	India	Bhattacharyya DR, Prakash A, Sarma NP, Mol
Anopheles	99.3253	14.4401	a village ne	Thailand	Kongmee M, Achee NL, Lerdthusnee K, Bang
Anopheles	104.5056	15.1418	Ubon Ratc	Thailand	Sumarnrote A, Overgaard HJ, Marasri N, Fus
Anopheles	106.27	17.49	Khe Ngang	Vietnam	Manh CD, Beebe NW, Van VN, Quang TL, Lei
Anopheles	106.4612	21.0203	Matiranga	Indian	Al-Amin HM, Elahi R, Mohon AN, Kafi MA, Ch
Anopheles	91.55	22.48	Thenzaw	Indian	Zomuanpuii R, Guruswami G, Nachimuthu Sk
Anopheles	92.45	23.19	Meghala	Indian	Srivastava AK, Kharbuli B, Shira DS, Sood A. E
Anopheles	89.51	25.32	north-east	Indian	Prakash A, Bhattacharyya DR, Mohapatra PK
Anopheles	91.48	26.08	Na Chalua	Thailand	Poolphol P, Harbach RE, Sriwichai P, Aupalee
Anopheles	105.1431	15.1322	Hang Chuc	Vietnam	Manh CD, Beebe NW, Van VN, Quang TL, Lei
Anopheles	92.5252	24.4332			Das NG, Gopalakrishnan R, Talukdar PK, Baru
Anopheles	92.45	11.44	in north-ea	Indian	Nandi J, Kaul SM, Sharma SN, Lal S. Anthropo
Anopheles	93.4645	26.0116	Boko PHC (	Indian	Nandi J, Misra SP, Rajagopal R, Narasimham
Anopheles	91.1645	23.5009	Tripura	Indian	Das SC, Bhuyan M, Baruah I, Talukdar
Anopheles	105.4619	16.423	Nong coun	Laos	Vilayvone Maniphousay,WANG Jian, D
Anopheles	106.3737	16.3117	Nong coun	Laos	Vilayvone Maniphousay,WANG Jian, D
Anopheles	103.42	21.48	Yunnan Lai	China	LARGE-SCALE SPATIAL NICHE CHARA

ita MC, Mohapatra PK, Singh S, Sarma DK, Mahanta J. Spatial distribution and molecular characteriza
ita MC, Mohapatra PK, Singh S, Sarma DK, Mahanta J. Spatial distribution and molecular characteriza
hapatra PK, Singh S, Sarma DK, Kalita MC, Mahanta J. Molecular evidence for the involvement of Ano
hapatra PK, Singh S, Sarma DK, Kalita MC, Mahanta J. Molecular evidence for the involvement of Ano
hapatra PK, Singh S, Sarma DK, Kalita MC, Mahanta J. Molecular evidence for the involvement of Ano
hapatra PK, Singh S, Sarma DK, Kalita MC, Mahanta J. Molecular evidence for the involvement of Ano
hapatra PK, Singh S, Sarma DK, Kalita MC, Mahanta J. Molecular evidence for the involvement of Ano
hapatra PK, Singh S, Sarma DK, Kalita MC, Mahanta J. Molecular evidence for the involvement of Ano
s MJ, Chowpongpang S, Prabaripai A, Charoenviriyaphap T. Seasonal abundance and distribution of A
tec B, Thanispong K, Chareonviriyaphap T, Corbel V. Status of insecticide resistance in Anopheles mo
n CT, Nguyen DV, Xuan TN, Ngoc AL, Cooper RD. Vectors and malaria transmission in deforested, rur
akma S, Lord JS, Khan WA, Haque R, Norris DE, Alam MS. Role of underappreciated vectors in malari
K. A three year study on distribution and ecology of Anophelines in Thenzawl, Mizoram, India. J Envir
ffect of land use and land cover modification on distribution of anopheline larval habitats in Meghala
, Mahanta J. Taxonomical observations on Anopheles philippinensis/nivipes group mosquitoes in no
K, Sattabongkot J, Kumpitak C, Srisuka W, Taai K, Thongsahuan S, Phuackchantuck R, Saeung A, Cha
n CT, Nguyen DV, Xuan TN, Ngoc AL, Cooper RD. Vectors and malaria transmission in deforested, rur
ah I. Diversity and seasonal densities of vector anophelines in relation to forest fringe malaria in dis
phily of Anophelines in Duars of West Bengal and other regions of India. J Commun Dis. 2000 Jun;32
MV. Present perspectives of malaria transmission in Boko area of Assam. J Commun Dis. 1993 Mar;2
PK. Mosquito survey in Tripura. Indian J Malariol. 1991 Jun;28(2):129-34. PMID: 1687
DENG Yan, ZHOU Hong ning.An investigation of species composition of adult mosqu
DENG Yan, ZHOU Hong ning.An investigation of species composition of adult mosqu
CTERISTICS OF MOSQUITOES IN RESIDENTIAL AREAS OF LANCANG RIVER BASIN, Y

tion of Anopheles nivipes and Anopheles philippinensis (Diptera: Culicidae) in north-east India. Acta
tion of Anopheles nivipes and Anopheles philippinensis (Diptera: Culicidae) in north-east India. Acta
pheles nivipes (Diptera: Culicidae) in the transmission of Plasmodium falciparum in north-eastern Ind
pheles nivipes (Diptera: Culicidae) in the transmission of Plasmodium falciparum in north-eastern Ind
pheles nivipes (Diptera: Culicidae) in the transmission of Plasmodium falciparum in north-eastern Ind
pheles nivipes (Diptera: Culicidae) in the transmission of Plasmodium falciparum in north-eastern Ind
pheles nivipes (Diptera: Culicidae) in the transmission of Plasmodium falciparum in north-eastern Ind
pheles nivipes (Diptera: Culicidae) in the transmission of Plasmodium falciparum in north-eastern Ind
nopheles larvae in a riparian malaria endemic area of western Thailand. Southeast Asian J Trop Med
squitoes in Ubon Ratchathani province, Northeastern Thailand. Malar J. 2017 Jul 25;16(1):299. doi: 1
al communities in north-central Vietnam. Malar J. 2010 Sep 16;9:259. doi: 10.1186/1475-2875-9-259
ia transmission in an endemic region of Bangladesh-India border. Parasit Vectors. 2015 Apr 1;8:195.
on Biol. 2014 Mar;35(2):369-76. PMID: 24665764.
ya, India. J Vector Borne Dis. 2013 Apr-Jun;50(2):121-6. PMID: 23995313.
rth-east India. J Commun Dis. 2004 Dec;36(4):264-70. PMID: 16506549.
ithong U. Natural Plasmodium vivax infections in Anopheles mosquitoes in a malaria endemic area o
al communities in north-central Vietnam. Malar J. 2010 Sep 16;9:259. doi: 10.1186/1475-2875-9-259
trict Sonitpur, Assam (India). J Parasit Dis. 2011 Oct;35(2):123-8. doi: 10.1007/s12639-011-0053-4. Ep
2(2):95-9. PMID: 11198404.
5(1):18-26. PMID: 8014435.
389.
itoes in residential areas of Nong county of Savannakhet province in southern Lao PD
itoes in residential areas of Nong county of Savannakhet province in southern Lao PD
JNNAN PROVINCE

Trop. 2012 Jun;122(3):247-54. doi: 10.1016/j.actatropica.2012.02.065. Epub 2012 Feb 25. PMID: 223
Trop. 2012 Jun;122(3):247-54. doi: 10.1016/j.actatropica.2012.02.065. Epub 2012 Feb 25. PMID: 223
lia. Ann Trop Med Parasitol. 2010 Jun;104(4):331-6. doi: 10.1179/136485910X12743554759948. PMI
lia. Ann Trop Med Parasitol. 2010 Jun;104(4):331-6. doi: 10.1179/136485910X12743554759948. PMI
lia. Ann Trop Med Parasitol. 2010 Jun;104(4):331-6. doi: 10.1179/136485910X12743554759948. PMI
lia. Ann Trop Med Parasitol. 2010 Jun;104(4):331-6. doi: 10.1179/136485910X12743554759948. PMI
lia. Ann Trop Med Parasitol. 2010 Jun;104(4):331-6. doi: 10.1179/136485910X12743554759948. PMI
lia. Ann Trop Med Parasitol. 2010 Jun;104(4):331-6. doi: 10.1179/136485910X12743554759948. PMI
Public Health. 2012 May;43(3):601-13. PMID: 23077839.
.0.1186/s12936-017-1948-z. PMID: 28743278; PMCID: PMC5526291.
. PMID: 20846447; PMCID: PMC2945362.
doi: 10.1186/s13071-015-0803-8. PMID: 25889228; PMCID: PMC4416289.

f northeastern Thailand. Parasitol Res. 2017 Dec;116(12):3349-3359. doi: 10.1007/s00436-017-5653-
. PMID: 20846447; PMCID: PMC2945362.
ub 2011 Jul 13. PMID: 23024492; PMCID: PMC3235386.

R. DOI：10.11853/j.issn.1003.8280.2019.06.018. http：//navi.cnki.net/knavi/Journal
R. DOI：10.11853/j.issn.1003.8280.2019.06.019. http：//navi.cnki.net/knavi/Journal

;74100.

;74101.

D: 20659394.
D: 20659395.
D: 20659396.
D: 20659397.
D: 20659398.
D: 20659399.

-1. Epub 2017 Oct 29. PMID: 29082435.

)etail?pcode=CJFD&pykm=ZMSK
)etail?pcode=CJFD&pykm=ZMSK
