# Supplementary material for: Risk Assessment of Anopheles philippinensis and Anopheles nivipes (Diptera: Culicidae) Invading China under Climate Change
Source: Biology (Basel). 2021 Oct 3;10(10):998. doi: 10.3390/biology10100998 (PMC8533129; doi:10.3390/biology10100998)
Supplement: Supplementary file 1 [file biology-10-00998-s001.zip › Table S4.pdf]

species	longitude	latitude
An. philipp	100.4583	22.04167
An. philipp	100.5417	14.04167
An. philipp	100.5417	18.54167
An. philipp	100.625	18.375
An. philipp	100.7083	14.625
An. philipp	100.875	14.625
An. philipp	100.9583	14.04167
An. philipp	101.2083	14.625
An. philipp	101.2917	6.291667
An. philipp	101.875	13.95833
An. philipp	101.875	14.125
An. philipp	101.875	14.20833
An. philipp	101.875	14.29167
An. philipp	102.0417	12.625
An. philipp	102.2917	16.625
An. philipp	102.375	16.625
An. philipp	103.375	15.04167
An. philipp	103.4583	21.45833
An. philipp	104.5417	15.125
An. philipp	105.125	15.125
An. philipp	105.4583	16.45833
An. philipp	106.2917	17.45833
An. philipp	106.375	16.29167
An. philipp	106.4583	21.04167
An. philipp	121.0417	12.375
An. philipp	124.7917	7.291667
An. philipp	124.7917	7.375
An. philipp	125.0417	11.125
An. philipp	89.54167	25.29167
An. philipp	91.125	23.54167
An. philipp	91.45833	26.04167
An. philipp	91.54167	22.45833
An. philipp	91.54167	25.375
An. philipp	92.45833	22.54167
An. philipp	92.45833	23.20833
An. philipp	92.45833	24.45833
An. philipp	92.54167	23.54167
An. philipp	92.54167	24.45833
An. philipp	93.375	27.04167
An. philipp	93.45833	26.04167
An. philipp	93.54167	24.45833
An. philipp	94.04167	25.375
An. philipp	94.375	25.45833
An. philipp	94.375	26.04167
An. philipp	94.54167	27.29167
An. philipp	97.20833	24.29167
An. philipp	98.875	18.54167
An. philipp	98.875	18.875
An. philipp	98.95833	18.79167
An. philipp	99.20833	18.95833

An. philipp	99.29167	14.45833
An. philipp	99.70833	8.208333
An. philipp	99.79167	18.95833
An. philipp	99.875	18.54167
An. philipp	99.875	8.458333
An. philipp	99.95833	18.70833
An. philipp	99.95833	18.79167
An. philipp	99.95833	18.95833
An. philipp	99.95833	9.541667
