# Supplementary material for: Risk Assessment of Anopheles philippinensis and Anopheles nivipes (Diptera: Culicidae) Invading China under Climate Change
Source: Biology (Basel). 2021 Oct 3;10(10):998. doi: 10.3390/biology10100998 (PMC8533129; doi:10.3390/biology10100998)
Supplement: Supplementary file 1 [file biology-10-00998-s001.zip › Table S6.pdf]

species	longitude	latitude
Anopheles	100.5417	14.04167
Anopheles	100.7083	14.625
Anopheles	100.875	14.625
Anopheles	100.9583	14.04167
Anopheles	100.9583	14.45833
Anopheles	101.0417	14.625
Anopheles	101.2083	14.625
Anopheles	103.375	15.04167
Anopheles	104.5417	15.125
Anopheles	105.125	15.125
Anopheles	106.4583	21.04167
Anopheles	125.0417	11.125
Anopheles	106.375	16.29167
Anopheles	105.4583	16.45833
Anopheles	89.54167	25.29167
Anopheles	91.125	23.54167
Anopheles	91.45833	26.04167
Anopheles	91.54167	22.45833
Anopheles	91.54167	25.375
Anopheles	92.45833	11.45833
Anopheles	92.45833	22.54167
Anopheles	92.45833	24.45833
Anopheles	92.54167	23.54167
Anopheles	92.54167	24.45833
Anopheles	93.375	27.04167
Anopheles	93.45833	26.04167
Anopheles	93.54167	24.45833
Anopheles	94.04167	25.375
Anopheles	94.375	25.45833
Anopheles	94.375	26.04167
Anopheles	94.54167	27.29167
Anopheles	98.375	18.45833
Anopheles	98.70833	18.45833
Anopheles	98.875	18.54167
Anopheles	98.875	18.875
Anopheles	98.95833	18.875
Anopheles	99.20833	18.95833
Anopheles	99.79167	19.125
Anopheles	99.875	18.95833
Anopheles	99.875	19.04167
Anopheles	99.95833	18.70833
Anopheles	99.95833	18.79167
